# The lockdown experience scale for students (LESS)

**DOI:** 10.1186/s12909-022-03858-x

**Published:** 2023-11-03

**Authors:** Ronán M. Conroy, Karen Fitzgerald

**Affiliations:** 1 School of Population Health Sciences, RCSI University of Medicine and Health Sciences, Dublin, Ireland; 2 RCSI University of Medicine and Health Sciences, Dublin, Ireland

**Keywords:** Wellbeing, Cabin fever, Lockdown, Covid-19, Students, Depression, Social isolation, Demotivation

## Abstract

**Background:**

The Covid-19 pandemic has resulted in many student populations learning online in lockdown. While the mental health consequences of lockdown are increasingly understood, the core features of ‘cabin fever’ – the experience of lockdown – are poorly described.

**Methods:**

We conducted a questionnaire survey of 649 undergraduate medicine and health sciences students. Item content was developed based on current literature and input from student representatives.

**Results:**

Mokken scaling identified seven questions that together formed a strongly unidimensional scale which comprised two domains: social isolation/cabin fever and demotivation / demoralisation. Scale scores were significantly associated with depression, self-rated mental health, impaired study efficacy and doomscrolling.

**Conclusions:**

The adverse effects of lockdown on student wellbeing appear to be driven to an important extent by an experience of isolation and demotivation that corresponds to narrative descriptions of cabin fever. In the foreseeable event of future pandemics, these experiences are a promising target for health promotion in students studying in lockdown.

**Supplementary Information:**

The online version contains supplementary material available at 10.1186/s12909-022-03858-x.

## Introduction

With an average of two viral diseases a year jumping the species barrier to humans [1] in tandem with the destruction of the last of the natural environment [2], the Covid-19 pandemic must be seen as a vital learning opportunity to develop mechanisms for preventing, mitigating and managing the further pandemics that will inevitably occur in the coming years [3]. One important element of this preparation must be developing an understanding of the specific effects of pandemics on mental health.

The current pandemic has had a wide-reaching effect on societies, with major changes to patterns of work, education, travel and socialising. From the outset, it was recognised that the pandemic would have a significant negative impact on mental health [4–7]. Early research produced two themes of immediate significance. The first documented the adverse effects on wellbeing, leading to the description of a syndrome increasingly referred to as Covid stress syndrome – a traumatic syndrome with symptoms of intrusive worrying thoughts, hypervigilance (including doomscrolling) and avoidance [8–10]. These findings led to the development of the Covid stress syndrome scale [11, 12].

The second theme was the assessment of risk perception. Risk perception is a major driver of health related behaviours [13, 14] and is therefore important in controlling the spread of infectious diseases. The development of specific scales to measure risk perception around Covid [15, 16] has been important in helping to understand evolving public health behaviours and to inform public health interventions. In a rapidly-evolving research field it is difficult to summarise the current state of research, however there have been several valuable overviews of the literature by Zavlis [17] and Cortez [18].

The institution of lockdown regimes across the world led to concern about the effects of isolation on mental health [19–23]. In education, the switch to online learning has led to a reduction in social contact and interaction, a diminished sense of engagement in a learning community, increasing loneliness and the emergence of the phenomenon of cabin fever [24]. Although cabin fever is not yet a well-defined entity, it describes the adverse effects of being confined to a limited space with or without others. Anecdotally, it is characterised by anxiety, unusual tiredness or sleepiness, irritability, moodiness, boredom, depression, or feeling of helplessness. It is akin to, but distinct from the concept of entrapment [25].

To date there has been one proposed scale measuring cabin fever by Cong and Rabbani [26] which was developed on the basis of descriptive accounts. However, to date there have been no data-driven attempts to identify the characteristics of cabin fever and the social isolation associated with lockdown.

In common with other schools and universities, the RCSI University of Medicine and Health Sciences adapted its learning delivery to online platforms in response to national lockdown requirements. Aware of the significant effect on student mental health reported in the numerous publications in the first months of the Covid-19 pandemic, we conducted a survey of our students as part of our ongoing student support programme.

The survey was a scoping survey, designed to provide information across a broad spectrum of areas of concern. For this reason, instead of using multiple validated scales to assess each domain – which would have created an unwieldy survey that was unlikely to engage student engagement – we instead tapped each area using one or two sentinel questions.

In this paper we report on the derivation of a short scale to measure the experience of social isolation, based on responses to the survey.

## Methods

The questionnaire items were derived from an extensive survey of the rapidly-accumulating literature on the effects of the pandemic on wellbeing. In the first iteration, we derived items related to two broad domains. The first was fear of Covid, characterised principally by symptoms seen in response to trauma: hypervigilance, avoidance, and intrusive thoughts, images and worries around the trauma [12, 27, 28]. The second domain of concern was risk perception around Covid-19 [15]. We were naturally concerned that students who feared losing out academically if they became ill might conceal symptoms or fail to report contact with a symptomatic individual. Consultation with student representatives through the Students Union resulted in the identification of two further important areas: social isolation and its effects, and positive experiences of meaning and purpose. Finally, a round of item prioritisation and selection, in consultation with student representatives, reduced the questionnaire from 35 to 19 closed questions and a twentieth item that asked for other positive or negative experiences that the questionnaire had not covered.

The relevant questions from the questionnaire is included in Additional file 1: Appendix 1, and the relevant data available in the supplementary materials. The global mental health item was the standard WHO item [29]. The depression item was adapted from the Beck Depression Inventory [30], maintaining the response options of the original scale but prefacing them with the question “Are you feeling sad?”. The effect on academic perceived academic efficacy was measured by asking “How has Covid affected your studies in the past month?”

The survey was carried out seven months from the start of national lockdown, during a phase in which all teaching was carried out online, indoor hospitality and entertainment venues were closed, and persons were confined to a 10 km radius of their domicile.

The survey invitation was circulated by email to all RCSI undergraduates by the RCSI Quality Enhancement Office, which acted as a gatekeeper. The invitation was followed by two reminders, one from each of the investigators. Each student received a unique random key to the survey that precluded multiple responses. The final response rate of 649 questionnaires represents approximately 25% of the undergraduate population.

Data were analysed with Stata release 16.1, R [31] and JASP. Stata was used for data preparation, cleaning and checking, and for regression modelling, in which robust standard errors were used throughout. The R package “mokken” [32] was used to conduct a Mokken scaling analysis on the scale properties. Mokken scaling is a nonparametric procedure that iteratively selects items from a pool based on Guttman criteria for a unidimensional scale. When no further items can be added without violating a threshold criterion for item acceptance the procedure stops [32, 33]. We set the scaling threshold for item acceptance at the recommended level of 0·3 [34].

JASP [35] was used to calculate Cronbach’s alpha and its Bayesian 95% credible intervals. JASP and R were used to examine the network structure of the items, the latter using the MPsychoR [36] and qgraph [37] packages. The item network was constructed using multidimensional scaling of dissimilarities, and goodness of fit of the two-dimensional representation of the items was checked using Sheppard plots and Kruskal’s stress index, which is zero when the items can be represented perfectly on two dimensions, and increases with increasing lack of fit. While there are no universally-agreed thresholds for stress, a value of ≤0·15 is taken as reflecting adequate dimensional representation – that is, that the distances between the nodes of the network are interpretable as relative distances between the variables.

The survey was conducted through the RCSI Quality Improvement Office which acted as a gatekeeper to ensure that data were anonymised at source before being passed to the researchers. Ethical approval was obtained from the RCSI Research Ethics Committee (Record ID: 212548897).

## Results

### Scale construction and visualisation

Mokken scaling identified seven questions that together formed a strongly unidimensional scale, with a Loevinger’s H coefficient of 0·41 (SE 0·02) and an alpha of 0·77 (95% Credible interval 0·74 to 0·80). Scale scores were calculated as summed item scores. Checks for goodness of scaling revealed no significant violations of monotonicity or invariant item ordering. The relevant questions and their response options are shown in Additional file 1: Appendix 1.

**Fig. 1:**
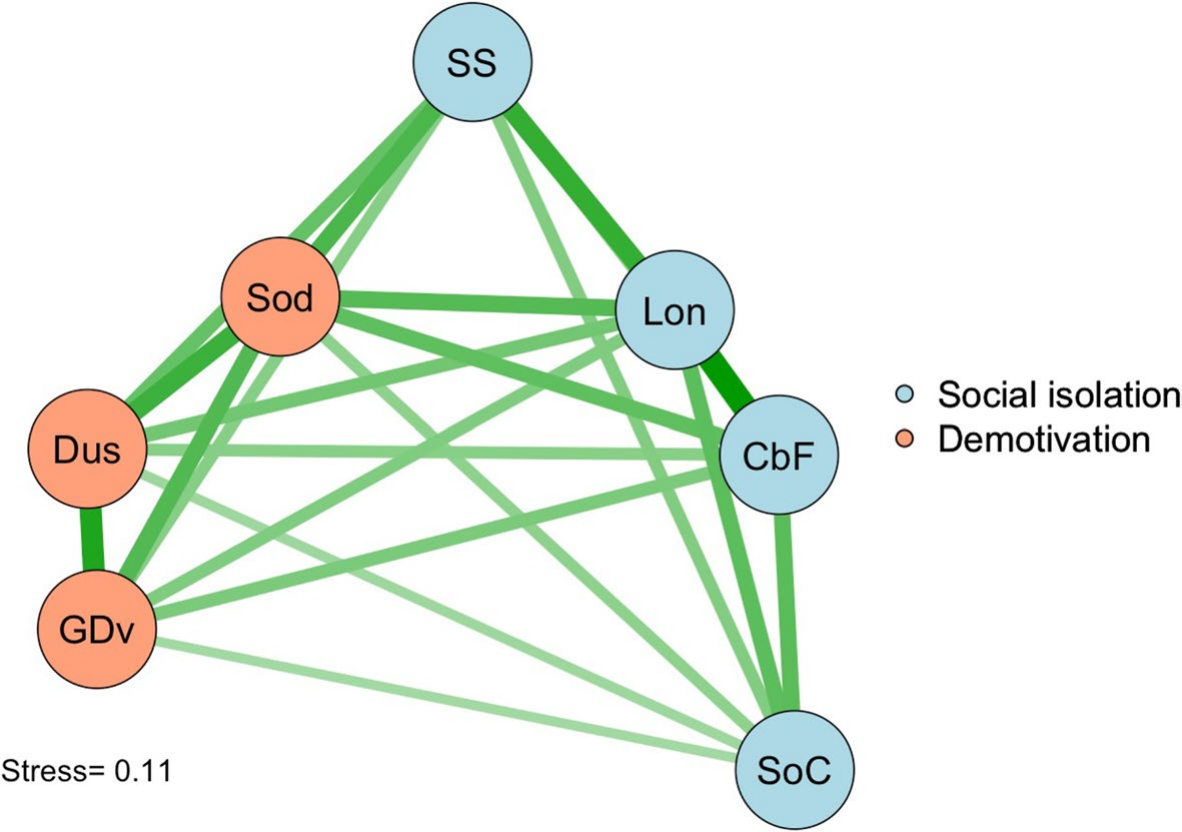
Network structure of the LESS items

Figure 1 shows the results of the network analysis of the item structure. We used multidimensional scaling of item dissimilarities to map the items onto a two-dimensional representation. We examined ordinal, spline and interval models of dimensionality. The diagram shows the spline model, which had a stress of 0·11 compared with 0·08 for the ordinal model, but lower than the 0·13 for the interval model. Examination of Shepard plots also revealed a consistent misfit for the interval model at intermediate item dissimilarities. The scaling in Fig. 1 is interpretable as the strength of the relationship between items. The items formed two groups. The first reflected a sense of social isolation – loneliness, lack of social support, cabin fever, a reduced sense of belonging to the learning community. The second reflected the loss of a sense of energy and purpose that we have termed demotivation. It comprised finding it hard to make a start on the day, doubting that you were doing the right thing and a lack of sense that you were growing and developing as a person.

**Table:** 

Abbreviation	Question on survey
SS	Do you have the support you need right now from friends and family?
Sod	How do you usually feel as you start the day?
Dus	Do you have a sense that you are doing something meaningful and worthwhile with your life?
GDv	Do you have a sense that you have really been growing and developing as a person in the past while?
Lon	Have you felt lonely in the last month?
CbF	Have you been suffering from “cabin fever” – feeling trapped at home a lot of the time
SoC	Has Covid affected your sense of being part of the [RCSI]* community?
	*this item may be changed in use to reflect the name of the school or community to which the participant belongs.

### Associations with scale scores

**Fig. 2:**
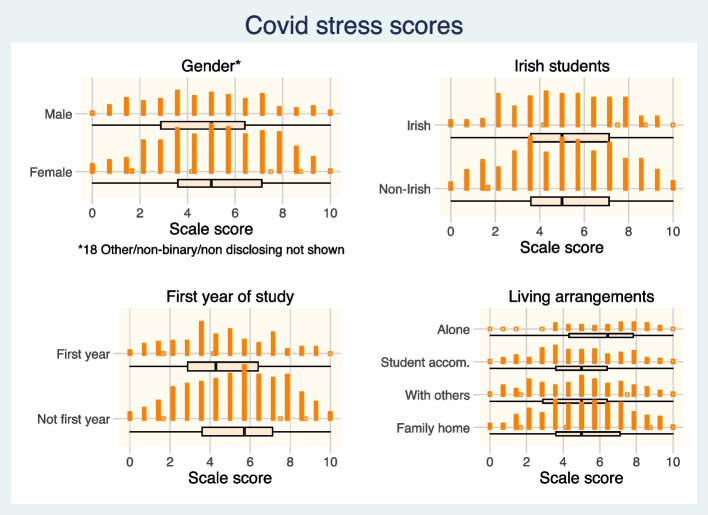
Lockdown Experience Scale for Students scores and demographic variables

Figure 2 shows mean scores on the Lockdown Experience Scale and demographic variables. Men students had a 56% probability of having a lower score than women students (Wilcoxon Mann-Whitney test, *P* = 0·027). Students in their first year had a 60% probability of having a lower scale score than students in subsequent years (Wilcoxon Mann-Whitney test, *P* < 0·001). Compared with students who were living in the family home, students who lived alone had higher scale scores (Ordinal logistic regression Continuation OR 2·2, 95% CI 1·3 to 3·9). Students in the other two residential categories – in shared accommodation or in student accommodation – did not differ in their scale scores from students who lived at home. Irish students, who might be expected to have more available family and social networks, had similar scores to students from other countries.

**Fig. 3:**
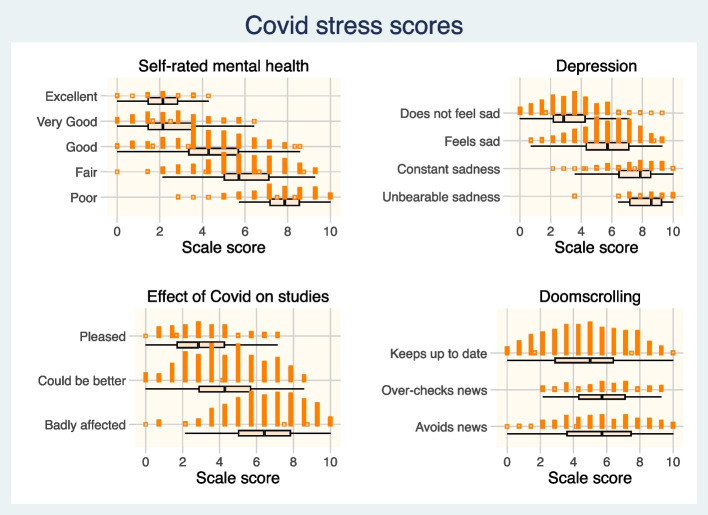
Lockdown Experience Scale for Students scores and wellbeing, academic efficacy

Figure 3 shows the relationship between Lockdown Experience Scale scores and variables of interest. Using ordinal logistic regression, there was a strong relationship between Lockdown Experience Scale for Students scores and both self-rated mental health (continuation OR for a 1-quartile increase in score 4·2, 95% Credible Interval 3·6 to 5·0) and depression (COR 4·4, 95% CrI 3·6 to 5·3). The association with traumatic behaviours showed that higher scale scores predicted both hypervigilance (doomscrolling) (relative risk ratio 1·4, 95% CrI 1·1 to 1·7) and avoidance of news (RRR 1·4, 95% CrI 1·1 to 1·6).

These associations remained substantially unchanged and statistically significant when region of origin, sex and course type were adjusted for (data not shown).

## Discussion

There has been considerable interest in the effects of the Covid-19 pandemic on mental health, with the proliferation of constructs and scales to measure these. These have been reviewed by Zavlis [17] and by Cortez [18]. The constructs fall into two broad groups: stress/distress, which includes anxiety, worry, fear and traumatic stress; and risk perception and health behaviours. There have been fewer attempts to examine the mechanisms whereby the pandemic brings about these effects. One logical place to begin is by examining the experience of isolation consequent on lockdown.

The effects of isolation are normally difficult to study in the general population because it is not the general population that typically experiences isolation. However, in an era that is increasingly characterised by epidemics and pandemics requiring populations to self-isolate, understanding these effects will become increasingly important to prevent and mitigate them.

The short scale presented here adds a potentially useful conceptual framework and measurement instrument to those available for examining the effects of social isolation on student mental health and morale, and adds to our understanding of the phenomenon of cabin fever. We freely admit that when we drew up the questionnaire the items that comprise the scale were selected to tap two domains that we considered distinct: social isolation and morale. However, as can be seen from the network analysis, these two domains have strong intersections, and the questionnaire items formed a unidimensional scale with a H coefficient of 0·41. This suggests that there is a superordinate construct that combines social isolation and demotivation.

This combination of isolation and demotivation corresponds well with the narrative accounts of the experience of cabin fever. These descriptions encompass both the sense of isolation and loss of the psychologically sustaining effects of social interaction, together with a sense of futility, demotivation, purposelessness [38–40]. It is useful to compare the items derived from the data-based approach used here with the items selected based on a literature review by Cong and Rabbani [26]. The twelve items in their scale loaded on two factors, one of anxiety, depression, hopelessness and anhedonia; the other reflecting changes in sleep and appetite. One item, decreased motivation, loaded with the sleep and appetite variables but had a substantial cross-loading with the first factor. The significant overlap between the scale items and the symptoms of depressive disorder makes it arguable as to whether the scale measures a specific construct of cabin fever.

The items in the LESS, on the other hand, form a single dimension having minimal conceptual overlap with either the constructs of depression (depressed mood and/or anhedonia) or anxiety (intrusive worry, panic, avoidance). As such it may more specifically represent the experience of lockdown.

LESS scores were associated with depression and poorer self-rated mental health. They were also associated with two manifestations of traumatic response: hypervigilance (doomscrolling) and avoidance. This suggests that response to isolation plays a significant role in wellbeing. Of course, we would not claim a clear direction of causation – indeed, it is probably unhelpful to model mental health variables as “outcomes”. Reciprocal reinforcing relationships can be expected to exist between all of the variables that we have presented. However, the greatest prospect for prevention and mitigation lies with the variables that represent the extent and quality of social interaction and social support. It is notable, but unsurprising, that scale scores were similar whether students were living in the family home, in student accommodation or in shared accommodation, with higher scores only in those who lived alone. Although we were unable to investigate the mental health effects of characteristics of the living environment in more depth, we would draw the reader’s attention to this under-researched area [40].

In common with most online surveys, the response rate (25%) deserves to be noted. While the prevalences reported in this paper are likely to be affected by non-response bias, the relationship that we have reported are less likely to be so influenced.

## Conclusion

Epidemics and pandemics are a predictable feature of the Anthropocene [1, 41]. We have been fortunate that the current pandemic has been comparatively benign compared with even recent events such as the early nineteenth century cholera pandemic or the influenza pandemic of the early twentieth. We must use the current learning opportunity not only to put in place preventive measures and plan co-ordinated response strategies, but also to learn how to maintain wellbeing and social cohesion in the context of lockdown and other restrictions to the daily life of the general public.

Lockdown is a distinctive form of social isolation, where the individual may be literally alone, or in permanent enforced proximity to a small group of individuals.^1^ It has brought dramatic changes to the delivery of learning and to the student experience. As educators, we need to understand the consequences of these changes. While we do know an increasing amount about the effects of lockdown on wellbeing, the concept and the experience itself remain poorly described and understood. We propose that the results presented here at least act as the basis for a more theory-driven approach.

### Supplementary Information

**Additional file 1: Appendix 1.** The LESS questions and response options

## Data Availability

A Stata dataset containing the variables reported in this paper is obtainable upon reasonable request from the corresponding author.

## Footnotes

1 One is tempted to remember Sartre’s play *Huis Clos*, in which the three protagonists discover that hell is not flames and torment, but being trapped in a drawing room forever with one another.
