# Supplementary material for: The lockdown experience scale for students (LESS)
Source: BMC Med Educ. 2023 Nov 3;23:829. doi: 10.1186/s12909-022-03858-x (PMC10625255; doi:10.1186/s12909-022-03858-x)
Supplement: Supplementary file 1 — Additional file 1: Appendix 1. The LESS questions and response options [file 12909_2022_3858_MOESM1_ESM.docx]

| **Question** | Response categories | Score |
| --- | --- | --- |
| **How do you usually feel as you start the day?** | I feel full of energy when I get up in the morning because I am looking forward to my day | 0 |
|  | I wouldn't go that far, but I'm generally pretty positive | 0 |
|  | Nothing special, just another day | 1 |
|  | I often find it really hard to motivate myself to get started on the day ahead | 2 |
| **Do you have a sense that you are doing something meaningful and worthwhile with your life?** | Yes, most days I really do | 0 |
|  | I wouldn't put it that strongly, but I feel I’m doing the right thing | 1 |
|  | No. I often wonder if I'm doing the right thing | 2 |
| **Do you have a sense that you have really been growing and developing as a person in the past while?** | Yes | 0 |
|  | I wouldn't put it that strongly, but yes – I feel I am developing as a person | 1 |
|  | No, I don't | 2 |
| **Have you been suffering from "cabin fever" – feeling trapped at home a lot of the time** | No | 0 |
|  | Yes, but not bad enough to affect my mood or studies significantly | 1 |
|  | Yes, and it's affected my mood and/or my studies | 2 |
| **Do you have the support you need right now from friends and family?** | Yes, my friends and family are a real support to me | 0 |
|  | I have less support than I'd really like, but I'm managing | 1 |
|  | I really miss the support of my friends and family" | 2 |
| **Have you felt lonely in the last month?** | No | 0 |
|  | Yes, but not enough to affect my mood or studies significantly | 1 |
|  | Yes, and loneliness has affected my mood and/or my studies" | 2 |
| **Has Covid affected your sense of being part of the [institution name] community?** | I have a stronger sense of really being part of the community these days | 0 |
|  | It's the same as before, more or less | 1 |
|  | I just don't have the same sense of being part of a community these days | 2 |
